# Supplementary material for: Magnetic domain walls as broadband spin wave and elastic magnetisation wave emitters
Source: Sci Rep. 2018 Sep 17;8:13871. doi: 10.1038/s41598-018-31689-8 (PMC6141534; doi:10.1038/s41598-018-31689-8)
Supplement: Supplementary file 1 — Supplementary information [file 41598_2018_31689_MOESM1_ESM.pdf]

Supplementary Material to:
Magnetic domain walls as broadband spin wave and elastic magnetisation wave emitters

Rasmus B. Holländer,[1] Cai Müller,[1] Julius Schmalz,[2] Martina Gerken,[2] and Jeffrey McCord[1]

[1]*Institute of Materials Science, Kiel University*

Kaiserstraße 2

24143 Kiel

Germany.

[2]*Institute of Electrical and Information Engineering, Kiel University*

Kaiserstraße 2

24143 Kiel

Germany.

Supplementary video 1 (Supplementary-video-1.mp4)

The pure in plane component Δm_{y} in a WDS is shown at an excitation frequency of 1.9 GHz. The timestamp for each frame is displayed at the bottom. The elastic magnetization waves are clearly visible in the centres of the domains. A strong uniform precession is also evident.

Supplementary video 2 (Supplementary-video-2.mp4)

The out of plane component Δm_{z} in a WDS is shown at an excitation frequency of 9 GHz. The timestamp for each frame is displayed at the bottom. The images are obtained by illumination in the polar configuration. The MSSW are visible in the centres of the domains.

Supplementary video 3 (Supplementary-video-3.mp4)

Time evolution of the Δm_{y} component from micromagnetic simulations at 9 GHz shown in Fig. 6c and 6d.

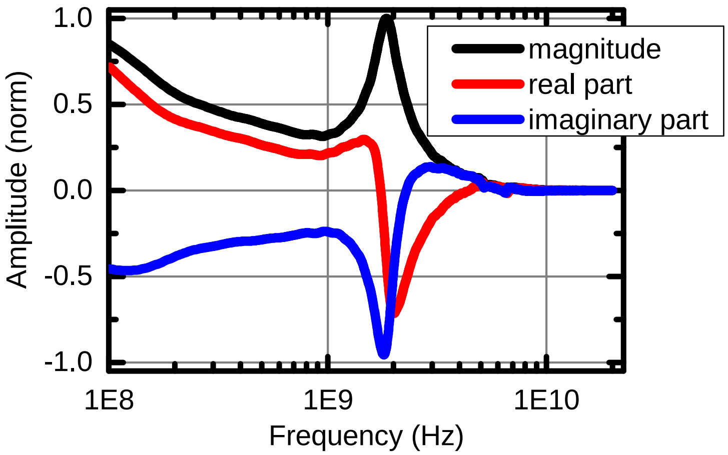

FIG. S1. Pulsed inductive microwave magnetometry (PIMM) spectrum of long amorphous $Co_{40}Fe_{40}B_{20}$ stripes with an easy axis of uniaxial anisotropy perpendicular to the long stripe axis. The axis of uniaxial anisotropy was aligned parallel to the center conductor of the coplanar waveguide. Before measuring the spectrum, the sample was initialized with the following field sequence: Saturating along the axis of uniaxial anisotropy and slowly reducing the field to zero. This field history is equivalent to the field history of the wide doimain state (WDS). A clear peak can be observed at 1.9 GHz indicating the resonance of the central domains of the WDS.
